# In vitro toxic evaluation of two gliptins and their main impurities of synthesis

**DOI:** 10.1186/s40360-019-0354-2

**Published:** 2019-12-19

**Authors:** Camila F. A. Giordani, Sarah Campanharo, Nathalie R. Wingert, Lívia M. Bueno, Joanna W. Manoel, Barbara Costa, Shanda Cattani, Marcelo Dutra Arbo, Solange Cristina Garcia, Cássia Virginia Garcia, Nádia Maria Volpato, Elfrides Eva Scherman Schapoval, Martin Steppe

**Affiliations:** 1 Laboratório de Controle de Qualidade Farmacêutico, Universidade Federal do Rio Grande do Sul (UFRGS), Porto Alegre, Brazil; 2 Laboratório de Toxicologia (LATOX), Universidade Federal do Rio Grande do Sul (UFRGS), Porto Alegre, Brazil

**Keywords:** Sitagliptin, Vildagliptin, Drug impurities, Cytotoxicity, Oxidative stress

## Abstract

**Background:**

The presence of impurities in some drugs may compromise the safety and efficacy of the patient’s treatment. Therefore, establishing of the biological safety of the impurities is essential. Diabetic patients are predisposed to tissue damage due to an increased oxidative stress process; and drug impurities may contribute to these toxic effects. In this context, the aim of this work was to study the toxicity, in 3 T3 cells, of the antidiabetic agents sitagliptin, vildagliptin, and their two main impurities of synthesis (S1 and S2; V1 and V2, respectively).

**Methods:**

MTT reduction and neutral red uptake assays were performed in cytotoxicity tests. In addition, DNA damage (measured by comet assay), intracellular free radicals (by DCF), NO production, and mitochondrial membrane potential (ΔψM) were evaluated.

**Results:**

Cytotoxicity was observed for impurity V2. Free radicals generation was found at 1000 μM of sitagliptin and 10 μM of both vildagliptin impurities (V1 and V2). A decrease in NO production was observed for all vildagliptin concentrations. No alterations were observed in ΔψM or DNA damage at the tested concentrations.

**Conclusions:**

This study demonstrated that the presence of impurities might increase the cytotoxicity and oxidative stress of the pharmaceutical formulations at the concentrations studied.

## Background

Drug toxicity is one of the main challenges for the pharmaceutical industry and it also contributes to late-stage failures, increased cost, and market withdrawals [1]. Nowadays, besides the toxicity of compounds present in drug formulations, attention is being paid to the presence of impurities [2–6].

Impurities in pharmaceutical products involve undesirable chemicals that remain in active ingredients or are developed during formulation and also through aging as degradation products [7, 8]. The presence of impurities is a significant problem in the synthesis of new compounds, since this process occurs in starting materials, solvents, intermediates and by-products [9].

Pharmacological and toxicological profiles are responsible for the safety of a drug, and adverse effects can be caused by the presence of impurities present in pharmaceutical products. Therefore, it is necessary to monitor and control impurities to ensure the quality and safety of pharmaceutical products [10].

Regulatory units are attentive to this issue and have been searching for different strategies to ensure the quality and safety of pharmaceutical preparations [11, 12]. According to ICH-Q3A (R2), there are reporting, identification, and qualification thresholds. The latter relates to the data acquisition and evaluation process that determines the biological safety of an impurity or a profile of impurities at a safe level [13]. The thresholds for qualification of impurities are based on individual drugs, therefore evidence of the presence of such impurities is important when they are related with adverse reactions in patients [11].

Toxicity tests are used to evaluate the potential of a chemical in causing harmful effects in experimental systems. The research can be carried out on the drug product or substance containing impurities or on isolated impurities [13]. Among these deleterious effects, there is the possibility of impurities that induce genetic mutations, breaks, and/or chromosomal rearrangements, with the potential to promote neoplastic alterations [14, 15]. Related to this, the European Medicines Agency (EMEA) published a guide in 2006 recommending that impurities should be identified concerning their genotoxicity or whether their chemical structure is an alert for toxicity. The concept of a toxicological concern threshold was also adopted, which establishes the safe dose for all potential carcinogens, corresponding to 15 μg per day [14–16]. The ICH M7 official guide determines the levels of impurities that are not carcinogenic and presents tests that evaluate the mutagenic control present in active substances or final products in order to ensure safety and quality for users [17].

Late complications of diabetes have been linked to hyperglycemia-induced oxidative stress. The mechanisms underlying hyperglycemia-mediated cellular damage include the formation of advanced glycation end-products, increased oxidative stress, mitochondrial dysfunction, and activation of the polyol and hexosamine pathways [18]. There is evidence that the main source of oxidative species production in diabetes is mitochondria [19]. Abnormal mitochondrial functions and excessive production of free radicals play a primary role in the onset of diabetes and its complications. Liver and kidney damage, which was partially reverted with n-acetylcysteine treatment, was observed in diabetic rats [20]. In humans, early kidney impairment was related to a hyperglycemia-induced oxidation process [21]. In this scenario, the presence of toxic impurities in therapeutic drugs may compromise treatment, aggravating disease complications.

At the moment, there are no studies reported in the literature regarding the toxicity of gliptin impurities. Therefore, the aim of this work was to study the toxicity of the drugs sitagliptin and vildagliptin and their main impurities of synthesis, using the mouse fibroblast 3 T3 cell line as an in vitro model, as well as some underlying mechanisms related to their toxicity. Cytotoxicity was evaluated through the MTT reduction and neutral red (NR) uptake assays. In addition, some mechanisms such as oxidative stress (oxidative species production), inflammation (nitric oxide), mitochondrial function, and genotoxicity were also evaluated.

## Material and methods

### Chemicals

Sitagliptin phosphate reference standard (99.5%), vildagliptin reference standard (99.5%), and 3-(trifluoromethyl)-5,6,7,8-tetrahydro -[1, 2, 4] triazolo [4,3-a]pyrazine-HCl (99.3%) (impurity S1) were supplied by Sequoia Research Products (Oxford, UK). O-benzylhydroxylamine hydrochloride (99.0%) (impurity S2), 2-pyrrolidinecarboxamide (98.0%) (impurity V1) and 3-amino-1-adamantanol (96.0%) (impurity V2) were supplied by Sigma-Aldrich (Brazil).

All chemicals were used as supplied. Stock solutions of sitagliptin, vildagliptin, and impurities S1, S2, V1, and V2 were made in purified water obtained from Millipore®. All stock solutions were stored at − 20 °C and freshly diluted on the day of the experiment.

### Cell culture

The 3 T3 cell line was routinely cultured in 75 cm^2^ flasks (Kasvi, São José dos Pinhais, PR, Brazil) using DMEM supplemented with 10% heat inactivated fetal bovine serum (FBS), 100 U mL^− 1^ penicillin (Gibco, Paisley, UK), and 100 mg mL^− 1^ streptomycin (Gibco, Paisley, UK). The cells were maintained at 37 °C in a humidified 5% CO_2_–95% air atmosphere. The cells were fed every 2–3 days, and sub-cultured once 70–80% confluence was reached.

### Cytotoxicity assays

The cytotoxicity was evaluated through the MTT reduction and NR uptake assays. The cells were seeded at a density of 3000 cells per well in 96 well plates. Triton X-100 1% (Sigma-Aldrich, St. Louis, USA) was used as a positive control. Negative control cells were incubated in culture medium. Concentration-response curves were obtained by incubating the cells with 0.5, 10, 50, 100, 500, and 1000.0 μM of sitagliptin, vildagliptin and their respective impurities for 24 h at 37 °C.

### MTT reduction assay

The MTT reduction assay was performed as previously described [22]. After 24 h of incubation of the cells with the drugs and impurities, the medium was removed and replaced by a fresh medium containing 0.5 mg mL^− 1^ MTT (Sigma-Aldrich, St. Louis, USA). The cells were incubated at 37 °C for 2 h. Afterwards, the cell culture medium was removed and the formed formazan crystals were dissolved in DMSO. The absorbance was measured at 550 nm in a multi-well plate reader (SpectraMax M2e, SoftMax® Pro 5, Molecular Devices, Sunnyvale, CA, USA). The results were graphically presented as percentage of MTT reduction vs. concentration (μM). All the drugs and impurities were tested in three independent experiments with each concentration tested in three replicates within each experiment.

### Neutral red uptake assay

The assay was performed according to OECD document 129 [23] and as previously described by Arbo et al. (2014) [18]. At the end of the 24 h of incubation-time of the cells with drugs and impurities, the medium was replaced by new medium containing 50 μg mL^− 1^ NR (Sigma-Aldrich, St. Louis, USA) and incubated at 37 °C for 3 h. After that, the cells were lysed with a 50% ethanol: 1% glacial acid acetic solution (v/v) (Sigma-Aldrich, St. Louis, USA). The absorbance was measured at 540 nm in a multi-well plate reader (SpectraMax M2e, SoftMax® Pro 5, Molecular Devices, Sunnyvale, CA, USA). The percentage of NR uptake relative to the control cells was used as the cytotoxicity measure. All the drugs were tested in three independent experiments with each concentration tested in three replicates within each experiment.

### Measurement of intracellular oxidative species

The intracellular oxidative species production was monitored by means of the DCFH-DA assay, as previously described [22]. For this determination, the cells were seeded at a density of 3000 cells per well in 96 well plates and allowed to grow for 24 h. On the day of the experiment, the cells were pre-incubated with 10 μM of DCFH-DA for 30 min at 37 °C in the dark. The cells were rinsed with PBS and incubated with the drugs and their impurities at 10, 100, and 1000 μM for 24 h. H_2_O_2_ (150 mM) was used as a positive control. Fluorescence was recorded in a fluorescence microplate reader (SpectraMax M2e, SoftMax® Pro 5, Molecular Devices, Sunnyvale, CA, USA) set at 485 nm excitation and 530 nm emission. The data obtained were calculated as the percentage of control conditions for each experiment from at least three independent experiments with each concentration tested in three replicates within each experiment.

### Measurement of nitric oxide

The cells were seeded at a density of 50,000 cells per well in 96 well plates and allowed to grow for 24 h. The drugs and their impurities were incubated at 10, 100, and 1000 μM for 24 h at 37 °C. After the incubation time, 100 μL of supernatant was transferred to another plate, 100 μL Griess reagent was added, and the plate was incubated at 37 °C. After 20 min of incubation time, the absorbance was measured at 540 nm in a multi-well plate reader (SpectraMax M2e, SoftMax® Pro 5, Molecular Devices, Sunnyvale, CA, USA). The data obtained were calculated as the percentage of control conditions for each experiment from three independent experiments with each concentration tested in three replicates within each experiment.

### Assessment of mitochondrial membrane potential (Δψm)

The estimation of Δψm contributes with important information about the mitochondrial function and also about the physiological state of the cell [24]. The evaluation of mitochondrial integrity was performed by measuring tetramethylrhodamine ethyl ester (TMRE) (Sigma-Aldrich, St. Louis, USA) inclusion as previously described [22]. The cells were seeded at a density of 3000 cells per well. After 24 h of incubation at 37 °C, the medium was gently aspirated and the cells were incubated with the drugs and their impurities at 10, 100, and 1000 μM for 24 h. Then, the medium was substituted by a new medium containing 2 μM of TMRE for 30 min at 37 °C in the dark. Afterwards, the medium was gently aspirated and replaced by phosphate buffer. Fluorescence was measured in a fluorescence microplate reader set to 544 nm excitation and 590 nm emission. The data obtained were calculated as the percentage of control conditions for each experiment from three independent experiments with each concentration tested in three replicates within each experiment.

### Comet assay

The cells were seeded in 12-well plates (Nest Biotech Co., Ltd., China) at a density of 200,000 cells per well. After 24 h, the medium was aspirated and the cells were incubated with the drugs and their impurities at 10, 100, and 1000 μM at 37 °C. After 24 h of incubation time, the cells were harvested by trypsinization (0.05% trypsin/EDTA). The cell suspensions were centrifuged (400×g, 5 min, 4 °C), the obtained cell pellets were resuspended in low-melting point agarose (0.75%, 150 μL) (Sigma-Aldrich (St. Louis, USA) and 60 μL aliquots were distributed on two slides coated with 1% normal-melting agarose. The samples were incubated in lysis solution (2.5 M NaCl, 100 mM EDTA, 10 mMTris–HCl, distilled water, 10% DMSO, and 1% Triton X-100) at 4 °C for 24 h in the dark. The slides were then incubated with alkaline electrophoresis running buffer (300 mM NaOH and 1 mM EDTA, pH 13) for 20 min at 4 °C before electrophoresis, which was carried out for 20 min at 25 V and 300 mA. After that, the slides were neutralized with 0.4 M Tris-HCL for 15 min in the dark. The DNA was fixed by immersing the slides in 70% ethanol for 15 min and in absolute ethanol for a further 15 min and left to dry overnight. For the microscopy analysis, the dried slides were stained with gel red (20 μg/mL) and DNA migration was observed in at least 100 cells at 400x magnification using a fluorescence microscope (Olympus, Japan) equipped with a 510–550 nm excitation filter of connected to a camera. The images were evaluated by Comet Score™ software, obtained from the public domain (http://www.tritekcorp.com/products_cometscore.php). The percentage of DNA in the comet tail (% DNA in tail) was the parameter evaluated to describe comet formation [25]. Concurrently with the comet assay, an extra and identical replicate comet slide was prepared, lysed, and immediately fixed and stained without electrophoresis for evaluation of the cytotoxicity using the low molecular weight (LMW) DNA diffusion assay [26].

### Statistical analysis

The results are presented as mean ± standard error of the mean (SEM) from at least three independent experiments. Normality of the data distribution was assessed by the Kolmogorov-Smirnov normality test. Significance was accepted at *p* < 0.05. Statistical comparisons between groups were performed by one-way ANOVA (when the data followed a normal distribution) or with the Kruskal-Wallis test (in the case of non-normal data distribution). Details of the statistical analysis are provided in the text and legend of the figures.

## Results and discussion

Sitagliptin and vildagliptin are used for the treatment of diabetes mellitus. They are well tolerated, with a low risk of hypoglycemia, they do not cause weight gain, and they are administered once a day [27]. The safety of pharmaceutical products should be considered, especially in chronic use where the daily accumulation of an impurity may compromise the patient’s health. The official guides recognize the importance of controlling drug impurities in order to limit human exposure; therefore, knowledge of the toxicity of impurities is necessary.

**Fig. 1:**
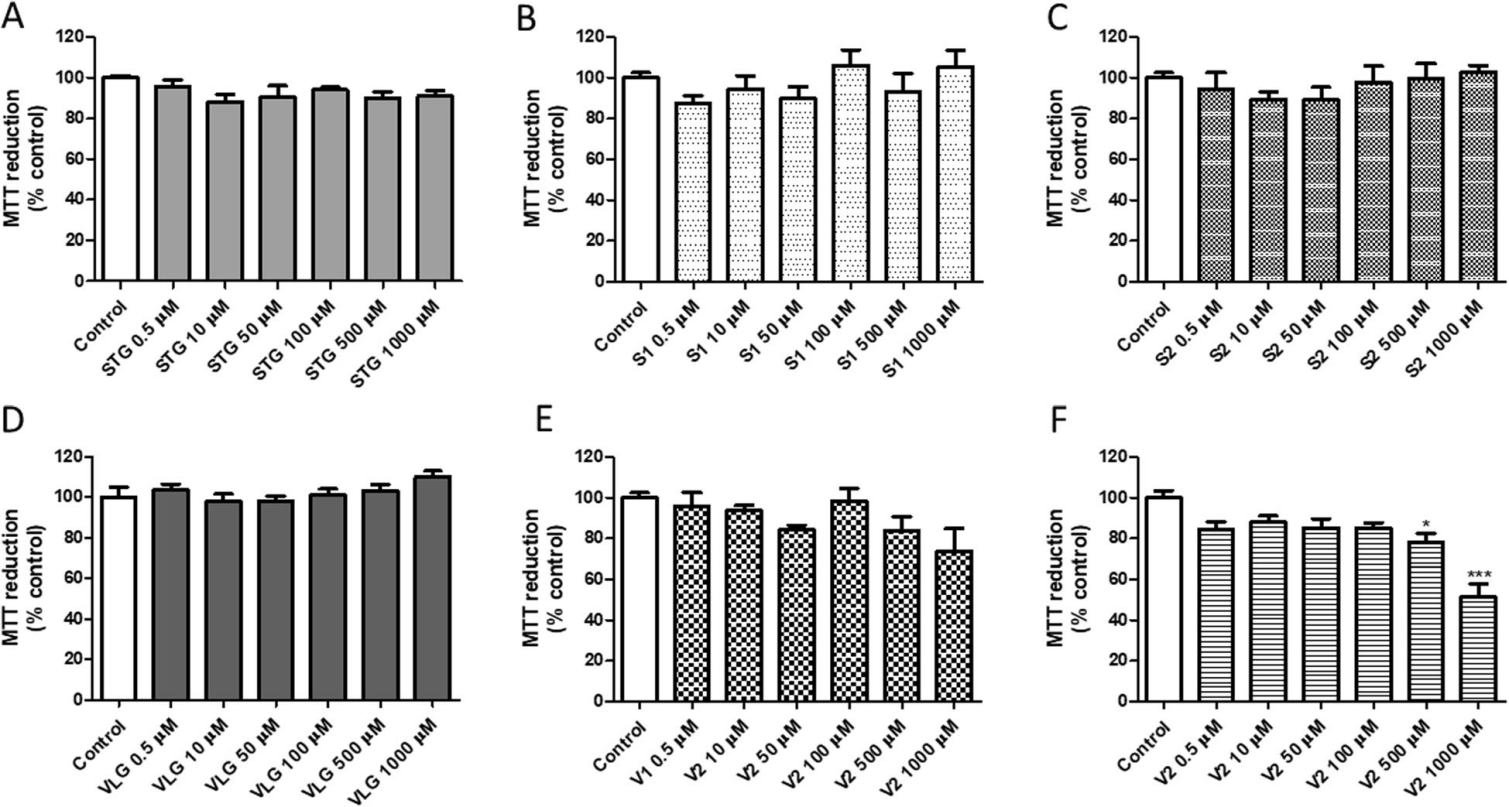
Cell viability evaluated by the MTT reduction assays in 3 T3 cells after 24 h incubations with: **a** sitagliptin – STG, **b** impurity S1, **c** impurity S2, **d** vildagliptin - VLG, **e** impurity V1, **f** impurity V2. Results are expressed as mean ± standard error of the mean. Statistical analysis performed through ANOVA/Bonferroni (*p < 0.05; ***p < 0.001 versus control)

As far as we know, this is the first study to investigate the toxicity of sitagliptin and vildagliptin, and their main impurities of synthesis. This is important because diabetes mellitus is a chronic disease related to oxidative stress and tissue damage such as diabetic nephropathy, diabetic neuropathy, and diabetic retinopathy. The presence of toxic impurities in drug formulations might compromise, or even worse the disease. The cytotoxicity analysis was carried out by incubating the 3 T3 cells with 0–1000 μM of sitagliptin, vildagliptin and their impurities for 24 h. The results obtained in the MTT reduction assay are presented in Fig. 1a-f. It was possible to observe a significant (*p* < 0.001, ANOVA/Bonferroni) decrease in MTT reduction at 500 and 1000 μM of V2 (Fig. 1f). No alterations in cell viability were observed after incubation of the 3 T3 cells with sitagliptin (Fig. 1a), impurity S1 (Fig. 1b), impurity S2 (Fig. 1c), vildagliptin (Fig. 1d) and impurity V1 (Fig. 1e). The assay evaluates the reduction of MTT tetrazolium salt (soluble in water) to formazan MTT (water insoluble) by cellular dehydrogenases within the metabolically active cells. This occurs when mitochondrial enzymes are active; correlating the number of viable cells with the increase in formazan production is used as an index of cell viability [28]. However, mitochondrial succinate dehydrogenase is susceptible to local modifications in ion concentrations and ion flux and a couple of chemicals that increase metabolic activity in a cell would result in increased mitochondrial succinate dehydrogenase activity [22].

**Fig. 2:**
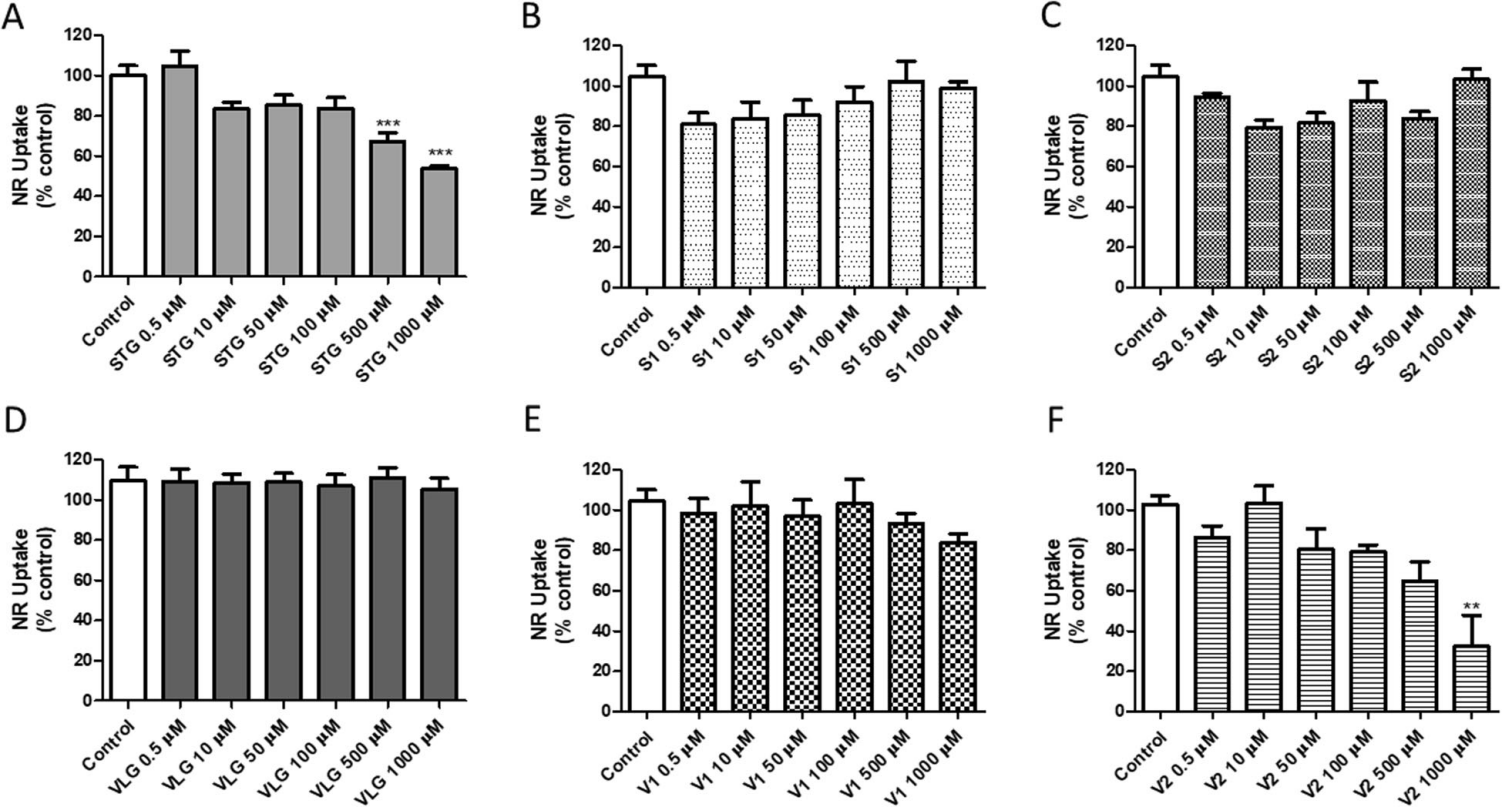
Cell viability evaluated by the neutral red uptake in 3 T3 cells after 24 h incubations with: **a** sitagliptin - STG, **b** impurity S1, **c** impurity S2, **d** vildagliptin - VLG, **e** impurity V1, **f** impurity V2. Results are expressed as mean ± standard error of the mean. Statistical analysis performed through ANOVA/Bonferroni (**p < 0.01; ***p < 0.001 versus control)

In the NR up-take assay (Fig. 2a-f), sitagliptin presented significant (*p* < 0.001, ANOVA/Bonferroni) cytotoxicity at concentrations of 500 and 1000 μM (Fig. 2a). In addition, impurity V2 also showed significant (*p* < 0.01, ANOVA/Bonferroni) decrease in cell viability at 1000 μM, the highest concentration (Fig. 2f), thus corroborating the results obtained with the MTT reduction assay. No alterations in cell viability were observed after incubation of the 3 T3 cells with impurity S1 (Fig. 2b), impurity S2 (Fig. 2c), vildagliptin (Fig. 2d) and impurity V1 (Fig. 2e). The neutral red uptake assay is based on the ability of the lysosomes of viable cells to incorporate the dye [29]. Interestingly, the results obtained by both tests generated slight variations, probably due to the use of different methods. This is not uncommon. Cadmium chloride (CdCl_2_) cytotoxicity was evaluated in HepG2 cells by MTT reduction, neutral red uptake, protein quantification, and LDH activity assays, and MTT reduction was shown to be more sensitive [30]. In contrast, in our research, the neutral red uptake assay was more sensitive compared to the MTT one for sitagliptin. Among the impurities, impurity V2 of vildagliptin showed toxicity through both MTT and neutral red assays.

**Fig. 3:**
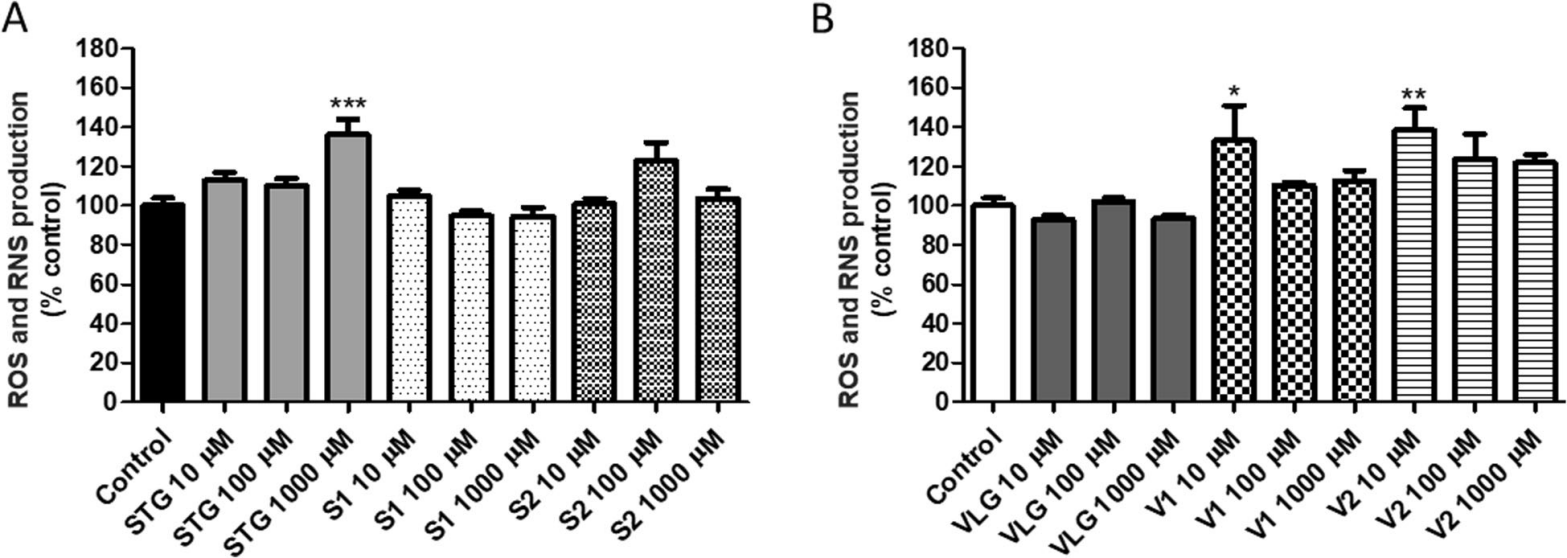
Production of reactive oxygen (ROS) and nitrogen (RNS) species in 3 T3 cells 24 h after incubation with DCFH-DA: **a** sitagliptin – STG and its impurities S1 and S2, **b** vildagliptin – VLG and its impurities V1 and V2. Results are expressed as mean ± standard error of the mean. Statistical analysis performed through ANOVA/Bonferroni (*p < 0.05; **p < 0.01; ***p < 0.001 versus control)

The effect of the drugs and their impurities on the generation of reactive species was evaluated by the DCFH-DA. This compound crosses cell membranes and it is enzymatically hydrolyzed by intracellular esterases in non-fluorescent dichlorodihydrofluorescein (DCFH). In the presence of oxidative species, this is oxidized to form a fluorescent compound (DCF) [31]. Figure 3a,b shows the results of the ROS and RNS production. A significant (*p* < 0.01, ANOVA/Bonferroni) increase in oxidative species was observed at 1000 μM of sitagliptin (Fig. 3a) and at 10 μM of impurities V1 and V2 (Fig. 3b). At high concentrations, free radicals can cause damage to lipids, proteins, and DNA, compromising the function of enzymes or transporters [4, 24]. Our results indicated an increase in the oxidative species production at 1000 μM of sitagliptin, suggesting that oxidative stress plays a role in its cytotoxicity. Increased reactive oxidative species were also observed for 10 μM of impurities V1 and V2. Considering that no cytotoxicity was observed at the same concentration level, it is supposed that compensatory mechanisms could be activated to counteract the free radicals at higher concentration levels.

**Fig. 4:**
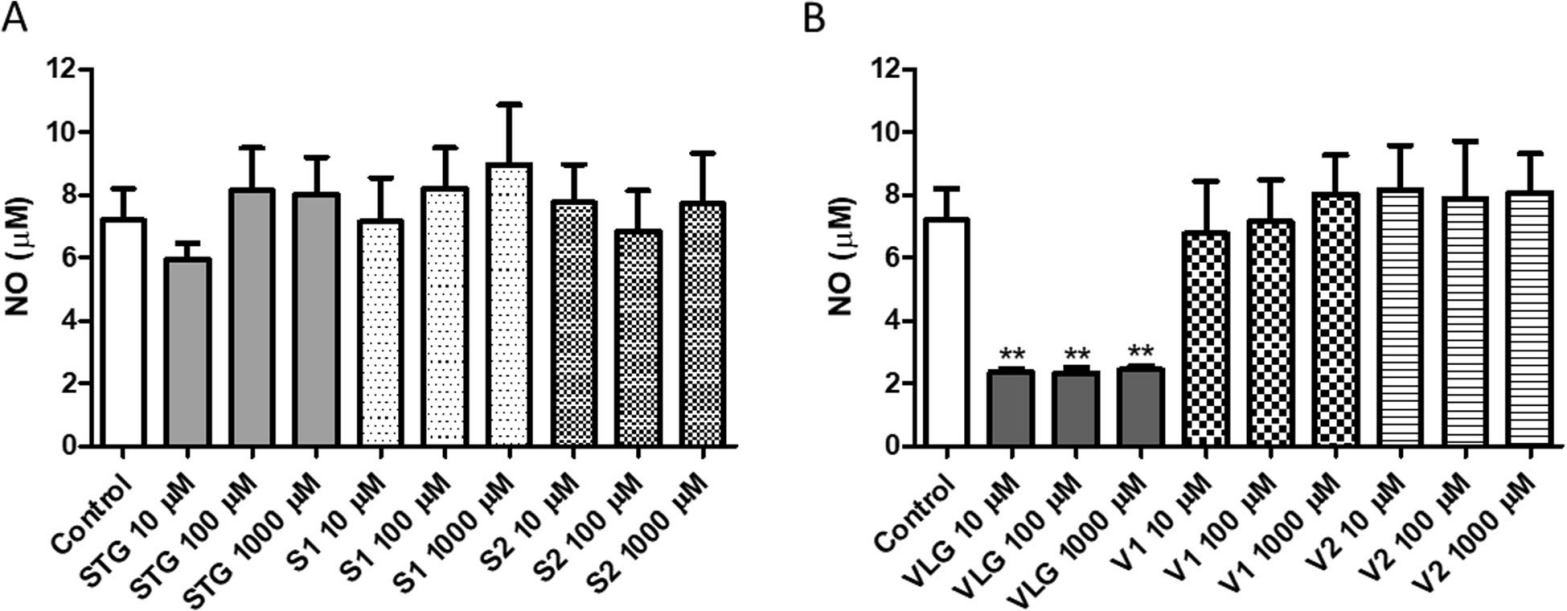
Evaluation of nitric oxide levels in 3 T3 cells 24 h after incubation with drugs and impurities: **a** sitagliptin – STG and its impurities S1 and S2, **b** vildagliptin – VLG and its impurities V1 and V2. Results are expressed as mean ± standard error of the mean. Statistical analysis performed through ANOVA/Bonferroni (*p < 0.05; **p < 0.01 versus control)

As depicted in Fig. 4a,b, the results obtained for NO production show that nor sitagliptin (Fig. 4a) or vildagliptin (Fig. 4b) impurities did not significantly alter the NO levels after 24 h of incubation. In cell culture conditions, NO formation from nitric oxide synthase accounts for the majority of nitrite, which is the major pathway for NO metabolism [32]. However, when there is an increase in reactive species, these free radicals may also mediate the endogenous formation of NO. This small molecule is related to chronic inflammatory diseases, playing an important role in the pathophysiology of different inflammation models [33]. The overproduction of NO from NO synthase and the activation of this enzyme by macrophages contribute to inflammation, cancer, diabetes and autoimmune disorders [34]. Interestingly, the antidiabetic drug vildagliptin presented a significant decrease in NO levels compared to the control (Fig. 4b). This result point to other beneficial effects of the drug in diabetes besides decreased glycemia levels and, the presence of the impurities in drug formulations could be detrimental to this effect.

**Fig. 5:**
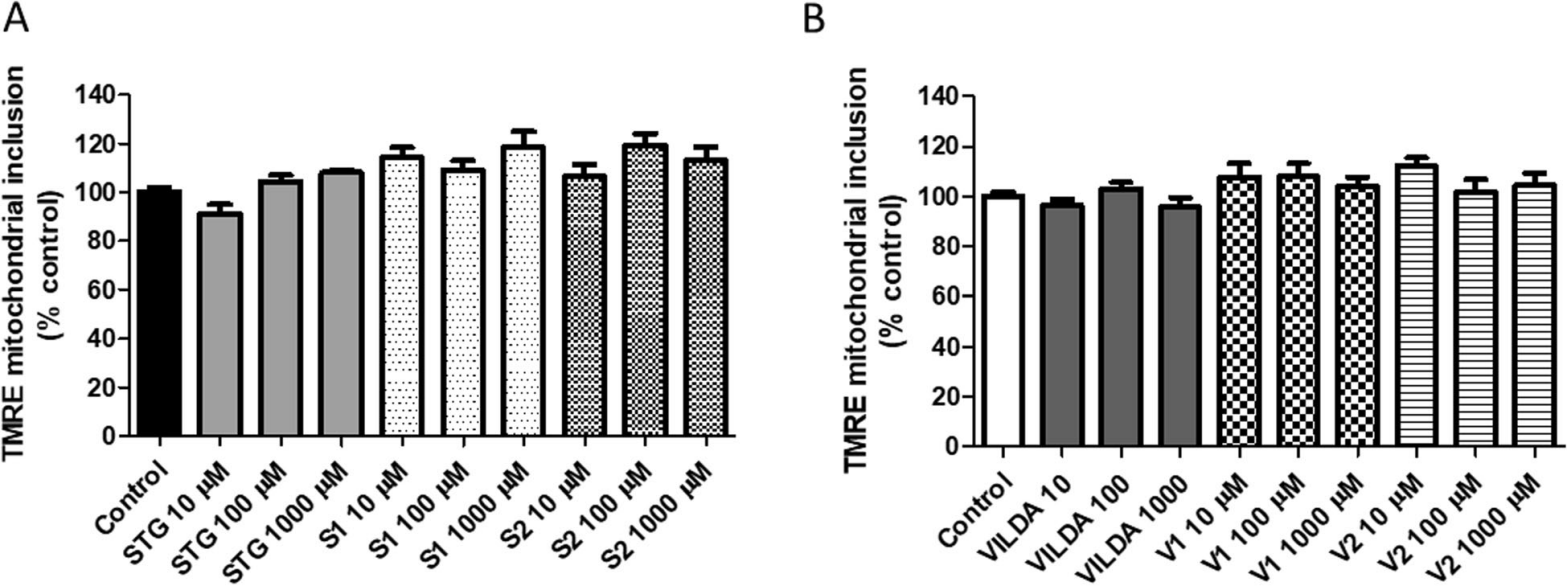
Evaluation of mitochondrial membrane potential (Δψm) in 3 T3 cells 24 h after incubation with drugs and impurities: **a** sitagliptin – STG and its impurities S1 and S2, **b** vildagliptin – VLG and its impurities V1 and V2. Results are expressed as mean ± standard error of the mean. Statistical analysis performed through ANOVA/Bonferroni

In order to investigate whether the compounds could disturb the mitochondrial function, the mitochondrial membrane potential was evaluated. In cells, mitochondria play an important role in normal function and are a regulator during the transition of cell death by both necrosis and apoptosis [35]. Δψm is responsible for controlling the accumulation of Ca^2+^ in the mitochondrial matrix, respiration and also the synthesis of ATP [36]. Because of its crucial role in the maintenance of the physiological function of the respiratory chain generating ATP, changes in Δψm compromise oxidative phosphorylation by reducing cell energy and inducing cell death [24]. As shown in Fig. 5a,b, no significant alterations were found in Δψm after 24 h of incubations of the 3 T3 cells with sitagliptin (Fig. 5a), vildagliptin (Fig. 5b), or their respective impurities.

**Fig. 6:**
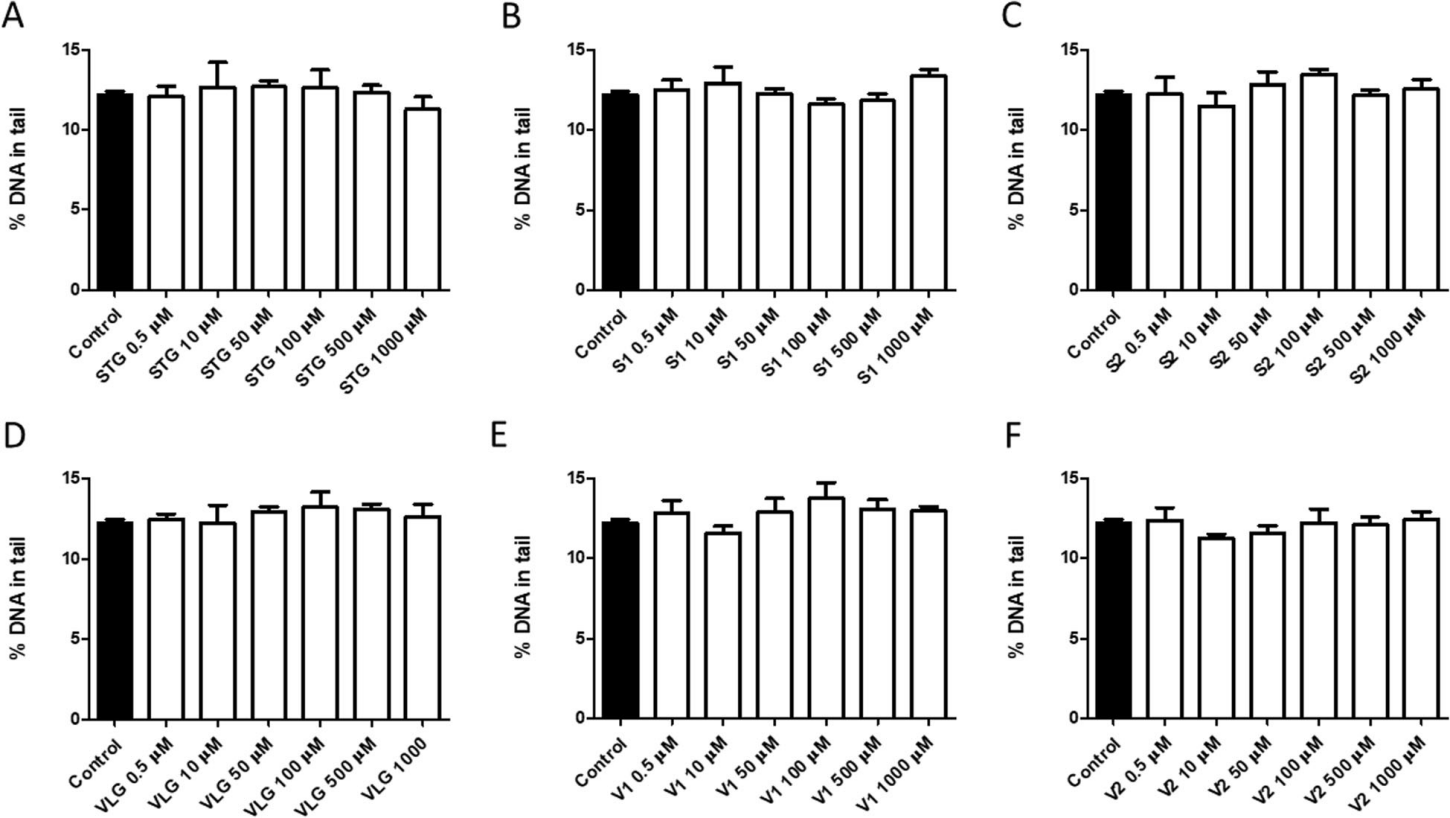
DNA damage in alkaline comet assay in 3 T3 cells 24 h after incubation with drugs and impurities. Sitagliptin – STG (**a**), impurity S1 (**b**), impurity S2 (**c**), vildagliptin – VLG (**d**), impurity V1 (**e**), impurity V2 (**f**). Results expressed as mean ± standard error of the mean. Statistical analysis performed through ANOVA/Bonferroni

**Fig. 7:**
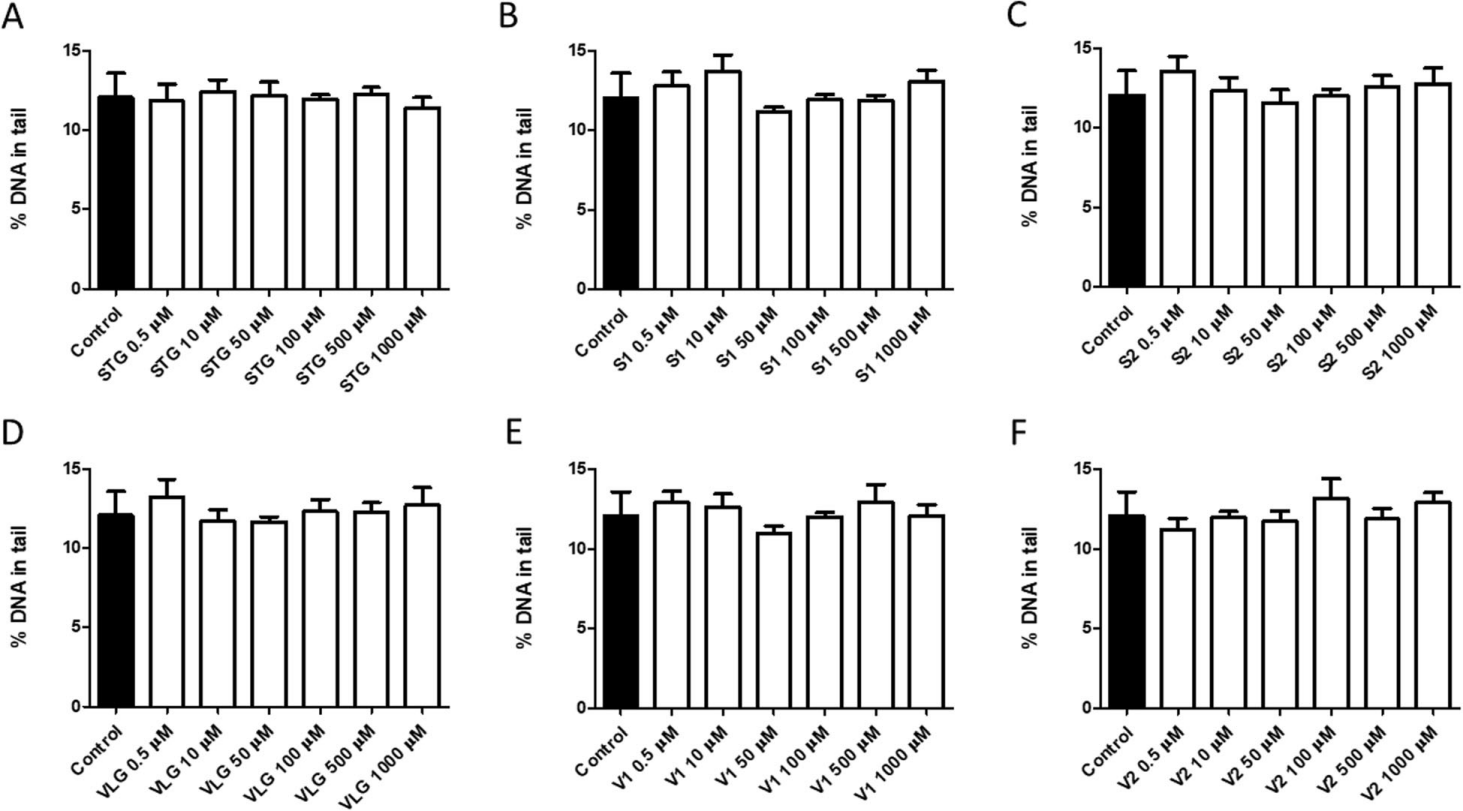
DNA damage in LMW DNA diffusion assay in 3 T3 cells 24 h after incubation with drugs and impurities. Sitagliptin – STG (**a**), impurity S1 (**b**), impurity S2 (**c**), vildagliptin – VLG (**d**), impurity V1 (**e**), impurity V2 (**f**). Results expressed as mean ± standard error of the mean. Statistical analysis performed through ANOVA/Bonferroni

The comet assay represents the capacity of negatively charged fragments of DNA to be extracted through an agarose gel in response to an electric field. It is a rapid, sensitive, and simple method for detecting DNA damage [37]. For this evaluation, the shape, size, and amount of DNA in comets are important for the test and correlate with the extent of DNA damage [37]. The results obtained by the alkaline comet assay indicate that neither sitagliptin (Fig. 6a), impurity S1 (Fig. 6b), and impurity S2 (Fig. 6c) nor vildagliptin (Fig. 6d), impurity V1 (Fig. 6e), and impurity V2 (Fig. 6f) elicited DNA breaks at the tested concentrations. The results of the LMW DNA diffusion assay indicated that, under our experimental conditions, neither sitagliptin (Fig. 7a), impurity S1 (Fig. 7b), and impurity S2 (Fig. 7c) nor vildagliptin (Fig. 7d), impurity V1 (Fig. 7e), and impurity V2 (Fig. 7f) induced significant cell death by apoptosis or necrosis.

## Conclusion

The safety of pharmaceutical products is important, mainly in chronic use due to their daily accumulation. In this case, the presence of impurities may compromise the patient’s health, and it is important to evaluate their toxicities. For the first time, the cytotoxicity of sitagliptin, vildagliptin and their chemical synthesis impurities were described in mouse fibroblast 3 T3 cells. Sitagliptin presented cytotoxicity at 500 and 1000 μM and increased oxidative species at 1000 μM but also decreased NO production at all concentrations. Moreover, except for impurity V2, the other impurities did not elicit significant cytotoxicity. This study provides important information to ensure the safety and quality of these drugs, which are available in the market. Furthermore, the presence of toxic impurities could be detrimental for diabetic patients, contributing to the tissue damage related to the progression of the disease and decreasing the therapeutic effect of the drugs.

## Data Availability

The data sets used and/or analyzed during the current study available from the corresponding author on reasonable request.
